# Comparative efficacy, safety and durability of dolutegravir relative to common core agents in treatment-naïve patients infected with HIV-1: an update on a systematic review and network meta-analysis

**DOI:** 10.1186/s12879-021-05850-0

**Published:** 2021-02-26

**Authors:** Katharina Nickel, Nicholas J. A. Halfpenny, Sonya J. Snedecor, Yogesh Suresh Punekar

**Affiliations:** 1 Pharmerit International, Berlin, Germany; 2 Pharmerit International, Rotterdam, Netherlands; 3 Pharmerit International, Bethesda, MD USA; 4 ViiV Healthcare, GSK House, 980 Great West Rd, Brentford, Middlesex TW8 9GS UK

**Keywords:** Antiretroviral therapy, Dolutegravir, HIV-1, Network meta-analysis, Systematic review, Treatment-naïve, Integrase strand inhibitors, Non-nucleoside reverse transcriptase inhibitor, Protease inhibitor

## Abstract

**Background:**

The objective of this study was to assess the durability of response of dolutegravir (DTG) as an antiretroviral core agent by comparing its efficacy and safety with other recommended or commonly used core agents up to 96-weeks (W96).

**Methods:**

A previously published systematic review was updated to identify phase 3/4 randomised controlled trials (RCTs) of core agents in treatment-naïve HIV-1 patients. Efficacy [virologic suppression (VS), CD4^+^ cell change from baseline] and safety [adverse events [AEs], discontinuations, drug-related AEs [DRAEs]] were analysed at W96 using Bayesian network meta-analysis (NMA) adjusting for nucleoside/nucleotide reverse transcriptase inhibitors' (NRTIs') backbone. Subgroups of patients with VL > 100,000 copies/mL or CD4^+^ ≤ 200 cells/μL at baseline were analysed separately.

**Results:**

The NMA included 20 studies reporting data at W96. A higher proportion of patients receiving DTG achieved VS compared to those on protease inhibitors [PI:Range:8.7%(CrI:3.1,16.0)-19.9%(10.8,30.5)], efavirenz [EFV:6.9%(1.3,10.8)] and cobicistat-boosted elvitegravir [EVG/c:8.2%(0.2,17.4)], and similar but numerically higher compared to rilpivirine [RPV:5.0%(− 2.8,12.5)], raltegravir [RAL:2.9%(− 1.6,7.7)] and bictegravir [BIC:2.7%(− 2.7,10.6)]. The probability that more patients on DTG would achieve VS at W96 compared to any other core agent was greater than 80%. A higher proportion of patients on DTG achieved VS compared to PI/rs [Range:33.1%(13.6,50.4)-45.3%(24.1,61.6)] and RAL [16.7%(3.3,31.2)] in patients with VL > 100,000 copies/mL at baseline, and similar VS was achieved in patients with CD4^+^ ≤ 200 cells/μL at baseline. DTG also achieved greater increase in CD4^+^ cells from baseline compared to EFV [32.6(10.7,54.7)], ritonavir-boosted darunavir [DRV/r:25.7(3.6,48.1)] and BIC [24.7(1.5,47.7)]. Patients receiving DTG had lower odds of discontinuing therapy by W96 compared to PI/rs, EFV, RAL and EVG/c. Patients on DTG had lower odds of experiencing an adverse event (AE) compared to patients on EFV [odds ratio:0.6(0.3,0.9)], ATV/r [0.4(0.3,0.6)] and LPV/r [0.3(0.2,0.5)]. For patients on DTG, the odds of experiencing a drug-related AE were lower than the odds for patients on EFV [0.3(0.2,0.4)], comparable to patients on RAL [1.1(0.8,1.4)] and higher than those on BIC [1.5(1.1,2.0)].

**Conclusion:**

Un-boosted integrase inhibitors had better efficacy and similar safety compared to PI/rs at W96 in treatment-naïve patients with HIV-1, with DTG being among the most efficacious core agent, particularly in patients with baseline VL > 100,000 copies/mL or ≤ 200 CD4^+^ cells/μL, who can be difficult to treat.

## Background

Human Immunodeficiency Virus type 1 (HIV-1) is a retrovirus that can lead to acquired immunodeficiency syndrome (AIDS), an advanced stage of HIV infection wherein the immune system is severely damaged. The advent of a multi-drug antiretroviral therapy (ART) has transformed HIV into a chronic condition with life expectancy comparable to that of the general population [1]. All the major guidelines recommend first-line ART composed of a core agent belonging to the integrase strand transfer inhibitors [INSTI] class in combination with one or two nucleoside/nucleotide reverse transcriptase inhibitors (NRTIs) [2–4]. European AIDS Clinical Society (EACS) guidelines also recommend specific core agents belonging to the ritonavir-boosted protease inhibitor [PI/r] class with two NRTIs as preferred regimen and specific core agents belonging to the non-nucleoside reverse transcriptase inhibitor [NNRTI] class with two NRTIs as an alternative regimen [3]. Overall, the most commonly used and recommended core agents include bictegravir (BIC), dolutegravir (DTG), cobicistat-boosted elvitegravir (EVG/c), and raltegravir (RAL) belonging to the INSTI class; atazanavir (ATV/r), darunavir (DRV/r), and lopinavir (LPV/r) belonging to the PI/r class; or efavirenz (EFV) and rilpivirine (RPV) belonging to the NNRTI class. These core agents, which differ in their efficacy, provide the antiretroviral strength to the combination allowing ARTs to vary in their ability to achieve and maintain virological suppression (VS). Furthermore, all classes of core agents are associated with tolerability and toxicity concerns with significant variations within each class [5]. It is therefore imperative to compare these agents in their efficacy, safety and durability to identify an ART suitable for appropriate patients.

Network meta-analysis (NMAs) allows comparison of individual core agents, based on a network of randomized clinical trial evidence, where head to head data are unavailable. A recent meta-analysis compared these core agents on efficacy outcomes such as VS and CD4+ cell count change from baseline, and safety outcomes including adverse events [AEs], discontinuations, discontinuation due to AEs and lipid changes [6]. Authors concluded INSTIs to have superior efficacy and comparable safety to PIs and NNRTIs with DTG being among the most efficacious INSTI in treatment-naïve HIV-infected patients. This study, however, was restricted to 48-weeks (W48), thus providing no evidence on the durability of these treatment effects. Another meta-analysis conducted in 2016 reported results up to 96-weeks (W96) but did not include newer core agents such as BIC [7]. The objective of our study was to compare DTG against other guideline-recommended core agents in treatment-naïve HIV-infected patients, to update our earlier work using evidence up to W96.

## Methods

A systematic search of PubMed/MEDLINE, Embase, and Cochrane databases was undertaken on July 19, 2019 to update the original search conducted in 2013 and updated in 2018 [6, 8]. Randomised controlled trials (RCTs) evaluating the efficacy and/or safety of ARVs in treatment-naïve people living with HIV (PLHIV) were identified. The search strategy for PubMed and Embase is available upon request. Further searches were conducted in the National Institute of Health clinical trial (NCT) registry database (www.clinicaltrials.gov). Additional records were identified through manual searching of article references. Two independent reviewers screened the study titles/abstracts to select studies which were further screened after reviewing full-text articles. Any discrepancies between the reviewers were resolved by consensus. Study data were extracted into a structured database by at least two independent reviewers and reconciled for accuracy. The Preferred Reporting Items for Systematic Reviews and Meta-Analyses (PRISMA) guidelines were followed in all phases of the study [9].

Studies were included if they were phase 3/4 RCTs of treatment-naïve adults or adolescents (≥13 years of age) with HIV-1 infection published in the English language. In addition, all studies were required to compare at least two of the core agents of interest in combination with two NRTIs and report at least one of the efficacy or safety outcomes of interest [6]. Studies investigating various dosage strengths of a core agent without an active comparator, with a sample size of less than 50 patients, or with paediatric populations (< 13 years of age) were excluded. The core agents included in the NMA were INSTIs (DTG, BIC, EVG/c, RAL), ritonavir-boosted PIs (ATV/r, DRV/r, LPV/r), and NNRTIs (EFV, RPV) [2–4].

The efficacy outcomes included in the NMA were the proportion of patients with VS at W96 and the change from baseline in CD4+ cell count at W96. In accordance with FDA guidance [10], VS was calculated as FDA Snapshot-50, time to loss of virologic response-50 (TLOVR-50), confirmed virologic response-50 (CVR-50), and HIV RNA < 50 copies/mL, and utilized within the NMA in that order of preference. The safety outcomes included were the proportion of patients with any AE, overall discontinuations, and drug-related AEs. In addition to the overall population, efficacy and safety outcomes were assessed in subgroups of patients with baseline viral load (VL) ≤100,000 and > 100,000, and patients with baseline CD4+ cell count ≤200 and > 200 cells/μL (secondary objective).

A Bayesian analysis framework was used to generate estimates of efficacy and safety of core agents relative to DTG [11, 12] using WinBUGS (version 1.4.3). Established frameworks were used to construct outcomes-based models [13]. For each outcome, a fixed-effect (FE) model and a random-effect (RE) model were evaluated. The Deviance Information Criterion (DIC) was used to select the model with a better fit between the FE and RE models. Further analyses were conducted to assess the heterogeneity in the treatment effects and inconsistency in the connected network.

Analyses were adjusted by the NRTI backbone combination included within each regimen. NRTI combinations were grouped into three categories: abacavir/lamivudine (ABC/3TC), tenofovir disoproxil (or alafenamide) fumarate/emtricitabine (TD[A]F/FTC), or any other NRTI combination (Other). Vague prior distributions (e.g. normal with mean 0 and variance 10^5^) on model parameters were used so that outcomes would be determined only by data from the RCTs. Posterior outcome distributions were based on at least 20,000 simulations after a burn-in of at least 10,000. Treatment effects for binary outcomes were modelled using binomial likelihood and identity (VS) or logit link function (AEs and discontinuations) to estimate the risk difference and odds ratios (OR) between the treatments. Treatment effect for changes in CD4+ cell count was modelled using a normal likelihood and identity link function to estimate the difference in the mean changes from baseline to W96 between the treatments of interest. Results were expressed as the median (50th percentile) of the posterior distribution of the treatment effect and 95% credible interval (CrI) – the 2.5th and 97.5th percentiles of the posterior distribution samples (i.e. representing the 95% probability that the parameter falls within this range). The Bayesian NMA methodology also allowed for estimates of the probability that one treatment is better than another to be calculated. As, by their nature, inferences from Bayesian analyses do not require adjustment for multiple comparisons [14], no adjustments for multiplicity were made.

## Results

**Fig. 1:**
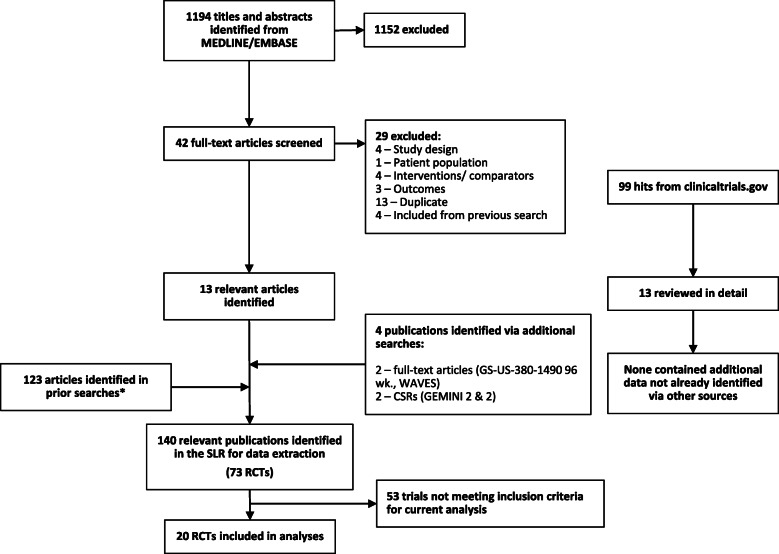
PRISMA chart

The systematic literature review identified a total of 1194 records from Medline and Embase databases and 100 records from clinicaltrials.gov (Fig. 1). The screening resulted in 1152 exclusions with a total of 42 full-text publications being assessed for data extraction. A further 4 records meeting the inclusion criteria were identified via secondary references. Of these, 29 articles were excluded at full-text review stage (Fig. 1). These 17 articles were added to the results of the previous SLRs, resulting in a total of 140 publications with 73 unique clinical trials [6]. Of these 20 studies conformed with the inclusion criteria and were included in the analyses [13–34].

**Fig. 2:**
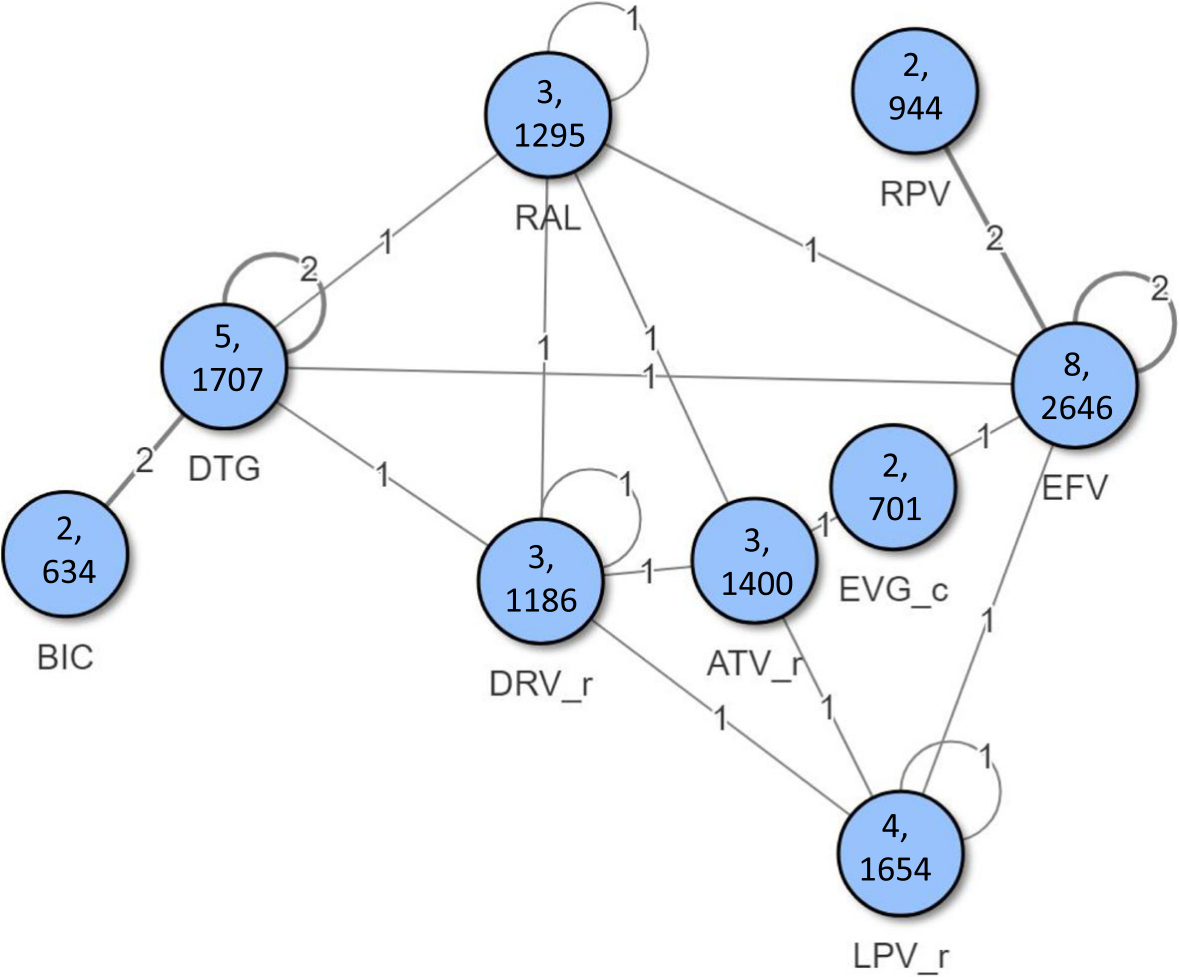
Network of treatment comparisons for efficacy outcomes. *Numbers inside node represent number of studies, number of patients for each core agent

The network of treatment comparisons for the efficacy outcomes is shown in Fig. 2. Based on model diagnostics the model with the lower DIC was used for the primary interpretation of efficacy and safety outcomes.

**Fig. 3:**
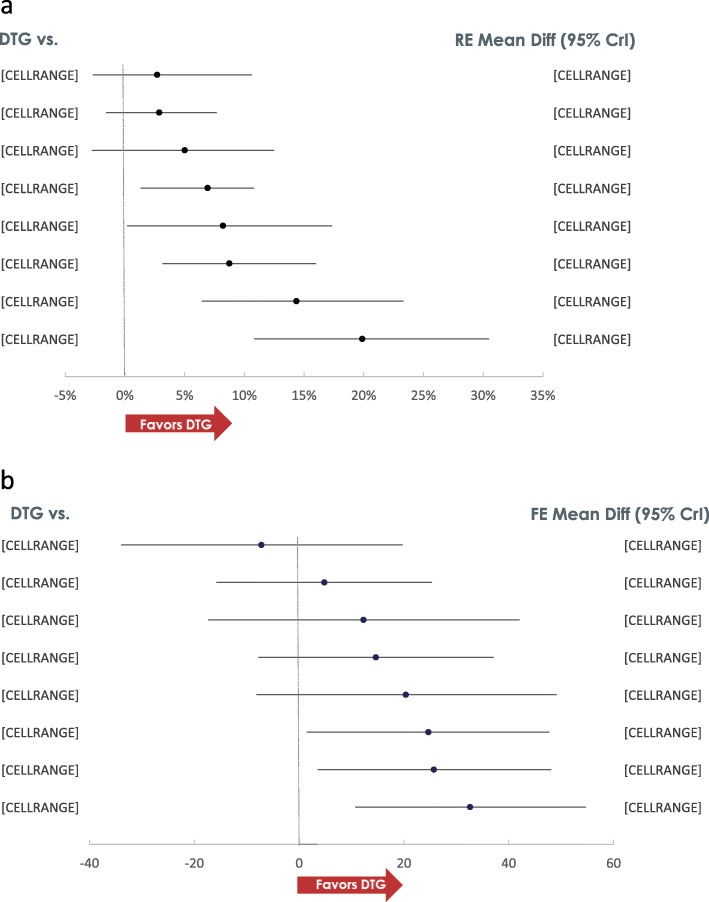
Efficacy Results. A: VS Risk Difference (RE model). B: CD4 difference (FE model)

At W96, higher proportion of patients receiving DTG achieved VS compared to all ritonavir-boosted PIs, EFV and EVG/c, and numerically higher but not statistically significant compared to RPV, RAL and BIC (Fig. 3). RAL and BIC were statistically superior to ATV/r [% Risk Difference (95% Credible Interval):11.5 (3.9, 19.9) and 11.6 (0.5, 21.9), respectively] and LPV/r [17.0 (8.0, 27.4) and 17.0 (5.3, 29.0), respectively], and EVG/c was superior to LPV/r [11.6 (0.8, 23.6)]. Among other core agents, DRV/r [11.0 (2.8, 20.1)], EFV [13.1 (3.6, 24.7)] and RPV [14.9 (3.6, 27.9)] were superior to LPV/r. The probability that treatment with DTG would result in more patients achieving and maintaining VS at W96 compared to any other core agent was greater than 80%. In patients with a high viral load at baseline (VL > 100,000 copies/mL), a higher proportion of patients on DTG achieved VS compared to DRV/r [33.1 (13.6, 50.4)], ATV/r [38.3 (13.3, 57.7)], LPV/r [45.3 (24.1, 61.6)] and RAL [16.7 (3.3, 31.2)], and similar proportions achieved VS compared to EFV [− 0.3 (− 15.4, 9.7)], RPV [2.5 (− 13.7, 14.7)], EVG/c [1.6 (− 18.1, 20.2)] and BIC [6.8 (− 7.9, 24.9)] at W96. EFV and BIC respectively, were superior to DRV/r [34.7 (9.3, 53.6); 25.8 (0.3, 49.3)], ATV/r [40.1 (10.2, 58.9); 30.8 (1.2, 56.0)] and LPV/r [47.3 (20.9, 61.8); 37.9 (11.2, 60.4)]. The results in patients with VL ≤ 100,000 copies/mL were comparable between all core agents with no statistically significant differences. A total of 4 studies in the network reported data on VS among patients with baseline CD4 ≤ 200 cells/μL at W96, resulting in only 4 core agents: RPV, EFV, DTG and BIC in the network. Results showed that in this subgroup, the VS achieved by patients on DTG was comparable to RPV [− 0.2 (− 66.0, 97.3)], EFV [10.8 (− 38.4, 30.9)] and BIC [16.8 (− 12.8, 49.3)]. Furthermore, the VS achieved by patients treated with RPV, EFV and BIC was comparable. Similarly, among patients with baseline CD4 > 200 cells/μL, the results were comparable between the four core agents with no statistically significant differences.

At W96, DTG achieved a greater increase in CD4 cell counts from baseline compared to EFV [32.6 (10.7, 54.7)], DRV/r [25.7 (3.5, 48.1)] and BIC [24.7 (1.5, 47.7)] and comparable changes to other core agents. Among other INSTIs, RAL achieved greater CD4 increases compared to DRV/r [20.9 (2.2, 40.0)] and EFV [27.8 (8.6, 46.9)]. The change in CD4 among patients treated with LPV/r was greater compared with DRV/r [32.9 (11.7, 53.9)] and ATV/r [21.8 (2.4, 41.3)].

No meaningful inconsistency was observed between the direct and indirect evidence of the VS network. However, in the CD4 network, differences were observed between the estimated outcomes of the 934 [25], ABCDE [26], and GS-US-380-1489 [19] studies. These differences were found to be attributed to differential results reported for the TDF/FTC and ABC/3TC backbones. Some studies reported little difference between these backbones (HEAT [34] and ASSERT [30]), the ACTG A5202 [28] trial also reported little difference when combined with ATV/r, but a difference of 30 cells/μL when combined with EFV.

### Safety

Patients receiving DTG had lower odds of discontinuing therapy by W96 compared to ritonavir-boosted PIs, EFV, RAL and EVG/c (Fig. 4). The all-cause discontinuations of DTG were similar to those among patients receiving BIC or RPV. The likelihood that fewer patients receiving DTG will discontinue therapy ranged from 86.8% vs RPV to 100% vs ritonavir-boosted PI therapies. Among INSTIs, patients treated with RAL had lower odds of discontinuations compared to EFV [odds ratio (95% CrI); 0.7 (0.6, 0.9)], DRV/r [0.7 (0.5, 0.9)], ATV/r [0.7 (0.6, 0.9)] and LPV/r [0.5 (0.4, 0.7)]. Patients treated with RPV also had lower odds of discontinuations compared to EFV [0.7 (0.5, 0.9)], DRV/r [0.7 (0.5, 0.9)] and LPV/r [0.5 (0.3, 0.7)] whilst LPV/r had higher odds of discontinuations compared to all core agents.

**Figure:**
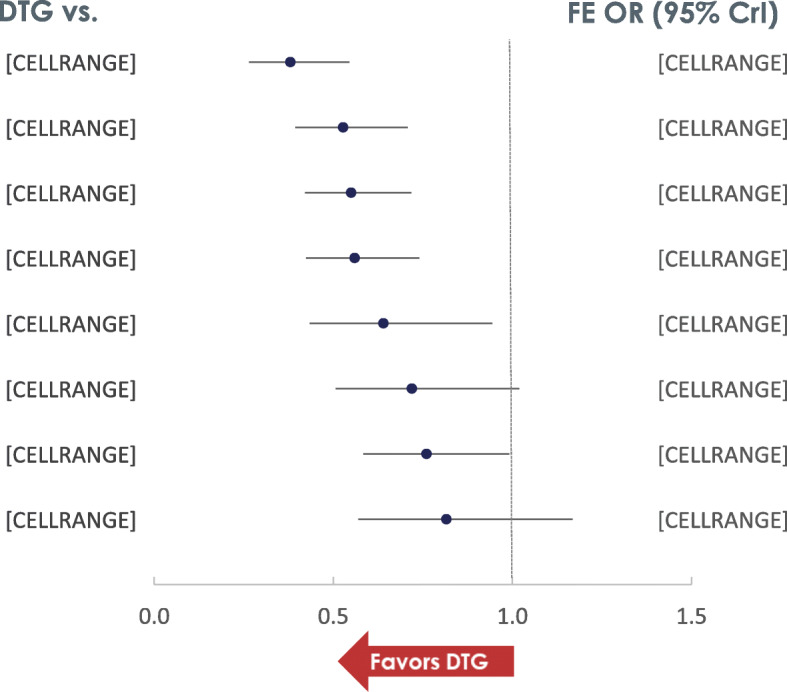
**Fig. 4** Odds of all-cause discontinuation (FE model)

Patients on DTG had lower odds of experiencing an adverse event (AE) compared to patients on EFV [0.6 (0.3, 0.9)], ATV/r [0.4 (0.3, 0.6)] and LPV/r [0.3 (0.2, 0.5)]. The odds were comparable against other core agents such as DRV/r [0.95 (0.7, 1.4)], RPV [0.8 (0.4, 1.6)], RAL [1.1 (0.8, 1.6)] and BIC [1.2 (0.8, 1.8)]. No data was available for EVG/c on AEs at week 96. Among other INSTIs, BIC and RAL, respectively had lower odds compared to EFV [0.5 (0.3, 0.9); 0.5 (0.3, 0.9)], ATV/r [0.3 (0.2, 0.6); 0.4 (0.3, 0.5)] and LPV/r [0.3 (0.1, 0.5); 0.3 (0.2, 0.4)]. Furthermore, DRV/r had lower odds of experiencing AEs compard to ATV/r [0.4 (0.3, 0.6)] and LPV/r [0.3 (0.2, 0.5)], and RPV had lower odds compared to LPV/r [0.4 (0.2, 1.0)].

Drug-related AEs were reported in 5 studies comparing 4 core agents: EFV, RAL, BIC and DTG. For patients on DTG, the odds of experiencing a drug-related AE were lower compared to EFV [0.3 (0.2, 0.4)], comparable to RAL [1.1 (0.8, 1.4)] and higher compared to BIC [1.5 (1.1, 2.0)]. Compared to EFV, the odds of experiencing a drug-related AE were lower for BIC [0.2 (0.1, 0.3)] and RAL [0.3 (0.2, 0.4)].

## Discussion

INSTI-based therapies are recommended as a preferred first -line treatments for PLHIV in all major guidelines [2–4]. Previous NMAs have concluded INSTIs, specifically DTG, to have higher odds of achieving VS at W48 compared to all ritonavir-boosted PIs and NNRTIs [6, 7]. Our previous NMA also indicated that higher proportions of patients receiving DTG achieve VS compared to all core agents up to W48 [6]. Results of this analysis suggest this trend to continue up to 96 weeks. In this analysis, higher proportions of patients treated with un-boosted INSTIs (DTG, RAL and BIC) were able to achieve and maintain VS up to 96 weeks compared to ATV/r, LPV/r, EFV and EVG/c, with DTG being numerically better than other core agents. The changes in CD4+ cell counts from baseline were also significantly greater or comparable for patients treated with DTG compared to all other core agents suggesting DTG as a core agent to be efficacious and durable. This was supported by consistent results in difficult to treat patients with high baseline viral load or low CD4+ counts. Furthermore, these benefits were achieved without any observed risk, such as discontinuations or AEs. These data suggest that INSTIs maintain better outcomes up to 96 weeks, with DTG being the most efficacious core agents available.

DTG is the most widely used ARV globally and is already recommended in guidelines as a 2- or 3-drug combination for treatment-naïve PLHIV. A recent NMA has established comparability of DTG + 3TC combination with guideline-recommended 3-drug regimen up to 48 weeks [35]. Despite this, there have been questions about the long-term efficacy of its effect, especially in the context of a 2-drug combination. Whilst we did not compare core agents as 2-drug combinations, our results suggest that DTG, as a core agent, provides a better platform compared to any other core agent to support a 2-drug combination. A DTG-based 2-drug regimen offers a realistic alternative for patients who want to reduce their ARV exposure. Additional long-term data on DTG-based 2DRs are needed to validate this hypothesis.

Evaluation of the quality of included studies was conducted previously for 48-week analyses where all trials were found to be of “strong” or “moderate” quality [6]. Most trials with “moderate” ratings were due to the common practice of unblinded treatments in this indication. Two trials are included in this analysis that were not evaluated previously [15, 26], both of which were moderate quality. Similarly, the quality of the NMA comparisons evaluated by the Grading of Recommendations Assessment, Development and Evaluation approach – although not repeated for the W96 data collection – are not expected to differ from that previously reported [36].

Consistent with our previous analyses, we combined data where any core agent was used in combination with TDF- or TAF-based NRTI-backbone. This was also essential to build the network with limited number of studies reporting data up to W96. This assumption of equivalence between TDF and TAF could be perceived as a limitation of these analyses. A previous study has found TAF to be non-inferior to TDF (both with EVG/c and FTC) in terms of VS, with similar safety profiles when compared in treatment-naïve patients with HIV-1 [37].

## Conclusion

Our systematic literature review and NMA provide further evidence to support the efficacy, safety and durability of INSTIs as the superior class of core agent for treatment-naïve patients with HIV-1 infection. It further reinforces DTG to be among the most durable first-line core agents up to W96, especially among difficult-to-treat patients, displaying a similar safety profile. DTG can be considered as a core agent of choice when considering reducing the treatment burden for appropriate patients.

## Data Availability

The datasets used and/or analysed during the current study are available from the corresponding author on reasonable request.

## Appendix 1

**Table 1:** Study characteristics and input data for 20 trials included in the NMA

Study (Source)	Treatment Arms	N	Male	Age, y	Baseline mean CD4+, cells/mL (SD)	Baseline mean viral load, log10 RNA copies/mL, (SD)	N with Virologic Suppression HIV RNA < 50 copies/mL (r/n)	CD4+ change, cells/μL	AEs (r/n)	Drug-related AEs (r/n)	Serious AEs (r/n)	Discontin-uations (r/n)
ACTG A5257 ([15])	ATV/r + TDF/FTC	605	76.20	37	309	4.60^a^	379/605	280	489/605	–	–	89/605
	DRV/r + TDF/FTC	601	76.21	37	310	4.61^a^	437/601	256	390/601	–	–	101/601
	RAL + TDF/FTC	603	75.46	36	304	4.66^a^	481/603	288	359/603	–	–	72/603
ECHO + THRIVE [16]	EFV + TDF/FTC	546	78.94	36	261^a^	5.0^a^	422/546	222	–	–	61/546	–
	RPV + TDF/FTC	550	78.00	36	247^a^	5.0^a^	423/550	226	–	–	52/550	–
FLAMINGO (CSR) [17]	DRV/r + ABC/3TC	242	83.06	34	400^a^	4.48^a^	60/80	274.4 (SD:226.64)	217/242	124/242	21/242	52/242
	DRV/r + TDF/FTC						104/162					
	DTG + ABC/3TC	242	87.19	34	390^a^	4.49^a^	65/79	298.2 (SD:199.95)	222/242	83/242	36/242	34/242
	DTG + TDF/FTC						129/163					
GS-236-0102 [18]	EFV + TDF/FTC	352	89.77	38	382 (170.2)	4.78 (0.6)	287/352	273	–	–	–	61/352
	EVG/c + TDF/FTC	348	88.22	38	391 (188.6)	4.73 (0.6)	293/348	295	–	–	–	53/348
GS-US-380-1489 [19]	BIC + TAF/FTC	314	90.76	31	443^a^	4.42^a^	276/314	287 (SD:207)	292/314	89/314	36/314	36/314
	DTG + ABC/3TC	315	89.52	32	450^a^	4.51^a^	283/315	288 (SD: 247)	302/315	127/315	39/315	31/315
GS-US-380-1490 [20]	BIC + TAF/FTC	320	87.50	33	440^a^	4.43^a^	269/320	237 (SD:204)	283/320	64/320	55/320	48/320
	DTG + TAF/FTC	325	88.62	34	441^a^	4.45^a^	281/325	281 (SD:209)	288/325	92/325	33/325	36/325
SINGLE (CSR) [21]	DTG + ABC/3TC	414	83.82	36	334.5^a^	4.67^a^	332/414	325.3 (SE:10.46)	376/414	184/414	44/414	72/414
	EFV + TDF/FTC	419	84.96	35	339^a^	4.7^a^	303/419	281.4 (SE:10.87)	394/419	282/419	51/419	109/419
SPRING-2 (CSR) [22]	DTG + ABC/3TC	411	84.67	37	359^a^	4.52^a^	125/169	292.2 (SD:195.70)	349/411	124/411	41/411	62/411
	DTG + TDF/FTC						207/242					
	RAL + ABC/3TC	411	86.37	35	362^a^	4.58^a^	124/164	286.2 (SD:192.45)	349/411	121/411	48/411	79/411
	RAL + TDF/FTC						190/247					
STaR (GS-US-264-0110) [23]	EFV + TDF/FTC	392	92.86	35	385 (187)	4.8 (0.6)	284/392	259 (SD:191)	368/392^b^	–	–	102/392
	RPV + TDF/FTC	394	92.89	37	396 (180)	4.8 (0.7)	307/394	278 (SD:189)	362/394^b^	–	–	78/394
STARMRK [24]	EFV + TDF/FTC	282	81.91	36.9	217.4 (133.6)	5 (0.6)	223/282	224.8	275/282	220/282	34/282	50/282
	RAL + TDF/FTC	281	80.78	37.6	218.9 (124.2)	5.0 (0.6)	228/281	239.6	266/281	132/281	40/281	36/281
934 study [25]	EFV + 3TC/ZDV	254	87.01	37	241^a^	5.0^a^	–	237 (SD: 136.4)	180/254	–	–	–
	EFV + TDF/FTC	255	85.88	36	233^a^	5.0^a^	–	270 (SD: 147.5)	185/257	–	–	–
ABCDE study [26]	EFV + ABC/3TC	115	74.8	38.4	203 (167)	4.94 (0.60)	70/115	263	–	–	–	40/115
	EFV + 3TC/d4T	122	78.7	38.5	223 (177)	4.92 (0.57)	58/122	294	–	–	–	59/122
ACTG A5142 [27]	EFV + CHOICE	250	81.20	39	195	4.8	158/178	230	–	–	–	–
	LPV/r + CHOICE	253	76.68	37	190	4.8	136/177	287	–	–	–	–
ACTG A5202 [28] (Clinicaltrials.gov)	ATV/r + ABC/3TC	463	83.80	38	236^a^	4.7 (0.7)	–	250.3^a^	–	–	–	141/463
	ATV/r + TDF/FTC	465	83.23	38.9	224^a^	4.7 (0.7)	–	251.5^a^	–	–	–	123/465
	EFV + ABC/3TC	465	78.92	38.4	225^a^	4.7 (0.7)	–	250.5^a^	–	–	–	141/465
	EFV + TDF/FTC	464	84.70	38.2	234^a^	4.7 (0.7)	–	220.5^a^	–	–	–	121/464
ARTEMIS [29]	DRV/r + TDF/FTC	343	69.68	36	228^a^	7.1^a^	271/343	171^a^	–	–	34/343	59/343
	LPV/r + TDF/FTC	346	69.65	35	218^a^	6.2^a^	246/346	188^a^	–	–	55/346	81/346
ASSERT [30]	EFV + TDF/FTC	193	79.79	36	230^a^	5.12^a^	76/128	220^a^	–	–	–	59/193
	EFV + ABC/3TC	192	82.81	38	240^a^	5.01^a^	57/112	235^a^	–	–	–	77/192
ATADAR [31]	ATV/r + TDF/FTC	90	86.67	35	328 (205)	4.8 (0.7)	–	284 (SD:219)	–	–	24/90	–
	DRV/r + TDF/FTC	88	88.64	37	341 (171)	4.8 (0.8)	–	298 (SD: 182)	–	–	7/88	–
CASTLE [32]	ATV/r + TDF/FTC	440	68.64	34	205	5.01	308/440	268	355/441	–	61/441	72/438
	LPV/r + TDF/FTC	443	68.62	36	204	4.96	281/443	290	370/437	–	48/437	95/440
GS-236-0103 [33]	ATV/r + TDF/FTC	355	89.01	39	366^a^	4.8 (0.62)	292/355	261	–	–	50/355	55/355
	EVG/c + TDF/FTC	353	91.78	38	351^a^	4.8 (0.61)	294/353	256	–	–	34/353	49/353
HEAT ([34], clinicaltrials.gov)	LPV/r + ABC/3TC	343	83.97	38	214^a^	4.903^a^	206/343	250^a^	274/343	–	42/343	109/343
	LPV/r + TDF/FTC	345	80.00	38	193^a^	4.844^a^	200/345	246.5^a^	276/345	–	45/345	124/345

^a^ Median was used instead of mean

^b^ Reported as treatment emergent adverse events, assumed to be equivalent to other reported AEs

## Abbreviations

ABC/3TC: Abacavir/lamivudine
AE: Adverse event
AIDS: Acquired immunodeficiency syndrome
ART: Antiretroviral therapy
ATV/r: Ritonavir-boosted atazanavir
BIC: Bictegravir
CD4^+^: Cluster of differentiation 4
CVR-50: Confirmed virologic response-50
DIC: Deviance Information Criterion DTG Dolutegravir
DRV/r: Ritonavir-boosted darunavir
EFV: Efavirenz
EVG/c: Cobicistat-boosted elvitegravir
FE: Fixed-effect
HIV-1: Human Immunodeficiency Virus type 1
INSTIs: Integrase strand inhibitors
LPV/r: Ritonavir-boosted lopinavir
NCT: National Institute of Health clinical trial
NMA: Network Meta-Analysis
NNRTIs: Non-nucleoside reverse transcriptase inhibitors
NRTIs: Nucleoside/nucleotide reverse transcriptase inhibitors
OR: Odds ratio
PRISMA: Preferred Reporting Items for Systematic Reviews and Meta-Analyses
PI/rs: Ritonavir-boosted protease inhibitors
PLHIV: People living with HIV
RAL: Raltegravir
RCTs: Randomised Controlled Trials
RE: Random-effect
RPV: Rilpivirine
TD[A]F/FTC: Tenofovir disoproxil (or alafenamide) fumarate/emtricitabine
TLOVR-50: Time to loss of virologic response-50
VS: Virologic suppression
W48: 48 weeks
W96: 96 weeks
