# Correction to: Comparative efficacy, safety and durability of dolutegravir relative to common core agents in treatment-naïve patients infected with HIV-1: an update on a systematic review and network meta-analysis

**DOI:** 10.1186/s12879-021-06016-8

**Published:** 2021-04-12

**Authors:** Katharina Nickel, Nicholas J. A. Halfpenny, Sonya J. Snedecor, Yogesh Suresh Punekar

**Affiliations:** 1 Pharmerit International, Berlin, Germany; 2 Pharmerit International, Rotterdam, Netherlands; 3 Pharmerit International, Bethesda, MD USA; 4 ViiV Healthcare, GSK House, 980 Great West Rd, Brentford, Middlesex TW8 9GS UK

**Correction to: BMC Infect Dis 21, 222 (2021)**

**https://doi.org/10.1186/s12879-021-05850-0**

Following publication of the original article [1], the authors identified errors in Figs. 3a, b and 4. The correct figures are given below.

**Fig. 3:**
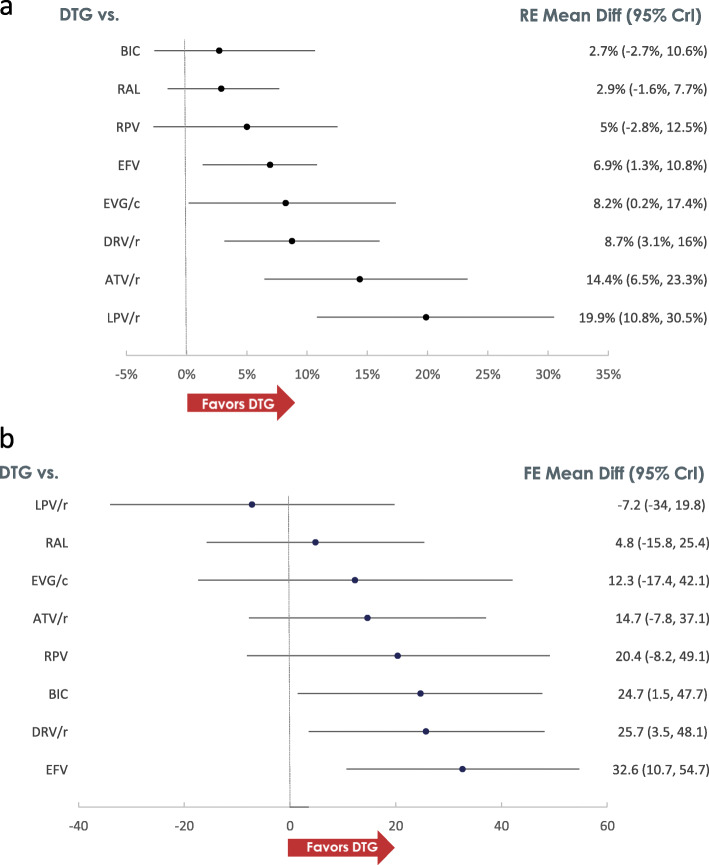
Efficacy Results. **a** VS Risk Difference (RE model). **b** CD4 difference (FE model)

**Fig. 4:**
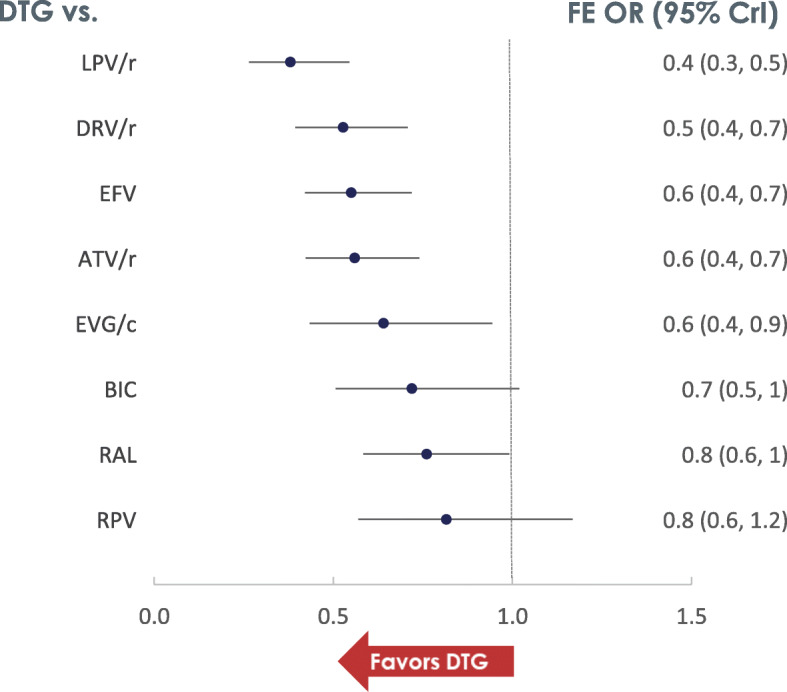
Odds of all-cause discontinuation (FE model)

The original article has been corrected.
